# Effects of pH, Temperature, Dissolved Oxygen, and Flow Rate on Phosphorus Release Processes at the Sediment and Water Interface in Storm Sewer

**DOI:** 10.1155/2013/104316

**Published:** 2013-11-18

**Authors:** Haiyan Li, Liang Liu, Mingyi Li, Xiaoran Zhang

**Affiliations:** ^1^Key Laboratory of Urban Stormwater System and Water Environment, Beijing University of Civil Engineering and Architecture, Beijing 100044, China; ^2^Department of Sustainable City Planning, Beijing Tsinghua Tong Heng Urban Planning and Design Institute, Beijing 100085, China

## Abstract

The effects of pH, temperature, dissolved oxygen (DO), and flow rate on the phosphorus (P) release processes at the sediment and water interface in rainwater pipes were investigated. The sampling was conducted in a residential storm sewer of North Li Shi Road in Xi Cheng District of Beijing on August 3, 2011. The release rate of P increased with the increase of pH from 8 to 10. High temperature is favorable for the release of P. The concentration of total phosphorus (TP) in the overlying water increased as the concentration of DO decreased. With the increase of flow rate from 0.7 m s^−1^ to 1.1 m s^−1^, the concentration of TP in the overlying water increased and then tends to be stable. Among all the factors examined in the present study, the flow rate is the primary influence factor on P release. The cumulative amount of P release increased with the process of pipeline runoff in the rainfall events with high intensities and shorter durations. Feasible measures such as best management practices and low-impact development can be conducted to control the P release on urban sediments by slowing down the flow rate.

## 1. Introduction

A great number of urban centers are drained by a unique sewer network in which wastewater is mixed with urban runoff water in wet weather [1]. Combined sewer overflows are major sources of intermittent pollution impacting the receiving water in many urban areas serviced by combined sewers [2]. Solid particles that cannot be transported at a certain hydraulic conditions can form deposits carried by wastewater and stormwater [3]. Furthermore, flushing of accumulated sewer sediment is one of the major sources of pollutants in urban wet-weather flow discharges [4].

Solids accumulated in sewer systems carry a variety of pollutants. Phosphorus (P), mainly present in sewage as orthophosphate [5], is one of the significant contaminants in sewer systems. Indeed, orthophosphate is known to quickly interact—uptake and release—with a wide variety of natural surfaces [6]. As an essential nutrient element, P can be utilized by microorganisms [7]. However, the release of P from the sediment in sewer threats water environment because of the eutrophication of water bodies. 

Focusing on the latter topic, a number of studies had paid attention to P release from the sediment to various kinds of receiving natural water bodies such as coastal zones [8], lakes [9], and rivers [10]. Some researches examined P release in urban catchment [11]. The release of P from sediments is a complex process [12]. Factors affecting the P release from the sediments have been extensively studied and reviewed. Previous studies [13] reported that the pH, dissolved oxygen (DO), and temperature at the sediment-water interface have a significant influence on the sediment P release, that is, anoxic levels and higher pH led to more P release into the water. However, few studies have been reported for sediments in storm sewer. Moreover, the relative research [14] showed that the content of soluble P in pore water in sediments is about 103 times than that in the overlying water. The P was flowed to the overlying water quickly under the disturbance. Therefore, the flow rate is a factor need to be studied on the P release at the sediments and water interface in storm sewer.

## 2. Materials and Methods 

### 2.1. Study Site

 North Li Shi Road is located in Xi Cheng District of Beijing, where drainage system is consisted of combined sewer system. The sampling site is located in a residential storm sewer of North Li Shi Road. The catchments are densely populated areas with many small retail shops and offices, but little industrial activity. The length of connection pipeline in the sampling site is 500 m with a gradient of 2~3‰. The diameter of connection pipe in the sampling site is 300 mm with 128 mm thickness of sediments. The environment temperature is the highest in July and August ranging from 20°C to 35°C, and 80% of the annual precipitation concentrated in the summer [15].

### 2.2. Sampling

The sampling was conducted in dry weather (the fifth consecutive sunny day after the heavy rain) on August 3, 2011. The sediment was taken from the storm sewer at a distance of 0.3–0.5 meters from the inspection well. In the pipeline, sediments with a 3–10 cm width of the cross section were sampled with a shovel. The stones and plastic were removed from the sample. They were then put in air-sealed plastic bags and taken to the laboratory. The samples were kept in 4°C iceboxes for further analysis [16]. 

After sampling the sediment, the samples of rainwater were collected directly in the sediment sampling point when the following rain occurred. The rainwater samples were kept in 4°C fridge for simulation of runoff scouring.

### 2.3. Effects of Environmental Factors on P Release at the Sediment and Water Interface

 The experiment was conducted in 1000 mL beakers with the overlying water at a depth of 6 cm. The samples were run in duplicates. The rainwater samples were filtered to remove the suspended solids and microorganisms [17]. The experimental facility was covered with a black plastic bag to avoid photosynthesis. In order to simulate the effects of pH on P release from sediments in storm sewer, the experiments were conducted at various pH of 4 ± 0.3, 6 ± 0.3, 7 ± 0.3, 8 ± 0.3, and 10 ± 0.3 at 26°C with 7 mg L^−1^ DO. NaOH and HCl were used to adjust the pH. To avoid the effect of ionic strength on P release, NaCl were added to control the salinity [18]. Effects of temperature on P release were studied at 15 ± 1°C, 20 ± 1°C, 25 ± 1°C, 30 ± 1°C and 35 ± 1°C in a biochemical incubator at pH = 8 and DO 7 mg L^−1^. Experiments for DO effects were conducted under the condition of 26°C and pH = 8. The DO were adjusted at 9 ± 0.3 mg L^−1^, 7 ± 0.3 mg L^−1^, 5 ± 0.3 mg L^−1^, and 3 ± 0.3 mg L^−1^ by adding Na_2_SO_3_. During the experiment, less than 1 mg L^−1^ DO was measured by the dissolved oxygen meters. The flow rate can be calculated using the corresponding rotational speed of the experiment. Five different flow velocity 0.3 m s^−1^, 0.5 m s^−1^, 0.7 m s^−1^, 0.9 m s^−1^, and 1.1 m s^−1^ were studied in the experiment, corresponding with the rotational speed of agitator 57 rmin^−1^, 96 rmin^−1^, 134 rmin^−1^, 172 rmin^−1^, and 210 rmin^−1^, respectively. The experiments were run at pH = 8, with temperature 26°C and DO 7 mg L^−1^. 

### 2.4. Analytical Methods

 The water level in these experiments was noted in order to keep the same water quantity after sampling and supplementation. 20 mL samples were extracted with a syringe for the analyses of total phosphorus (TP) in every 10 min. Then, an appropriate amount of rainwater was added to compensate for the loss of water and evaporation. For TP analysis, a water sample was autoclaved at 121°C for 30 min after K_2_S_2_O_8_ was added. Then, 1 mL ascorbic acid and 2 mL molybdate were added and the sample measured using the molybdenum-antimony antispectrophotometric method [19].

Because an appropriate volume of water sample was collected from the experimental apparatus, and clean water without P was supplied to the experimental apparatus, the cumulative release amount on the *n*th sampling is described as

(1) $$\begin{array}{c} R_{n}=V\left(C_{n}-C_{0}\right)+\sum_{j=1}^{n}V_{j-i}\left(C_{j-i}-C_{0}\right), \end{array}$$

where *V* is the volume of the overlying water, *C*_0_, *C*_*j*−1_, *C*_*n*_ is the P concentration in the overlying water on the first sampling, (*j* − 1)th sampling and *n*th sampling, respectively, and *V*_*j*−1_ are the volume of the sampling water. The phosphorus (P) loading in different environmental factors is described as

(2) $$\begin{array}{c} W=\frac{\left(R_{i}-R_{i-1}\right)}{ts}, \end{array}$$

where *R*_*i*_, *R*_*i*−1_ are the cumulative release amount on the *i*th sampling and (*i* − 1)th sampling, respectively, *t* is the time of sample interval, and *s* is the contact area at the sediment and water interface.

## 3. Results and Discussion

### 3.1. Sediment Characteristics

 The physicochemical properties were analyzed, including size fraction distribution (Table 1) and concentrations of various forms of P (Table 2).

### 3.2. Effects of pH on P Release from the Sediment and Water Interface

**Figure 1:**
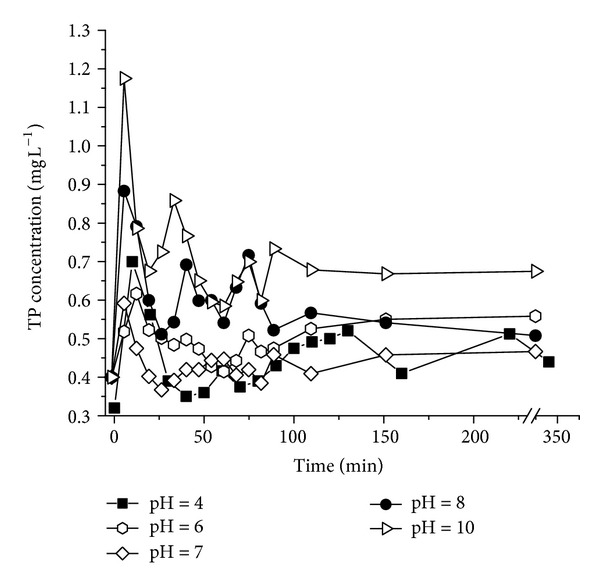
Concentration changes of TP in the pH effect experiments.

 Changes in P concentration as a function of pH in the release experiments are shown in Figure 1. The results suggest that P release from the sediments occurred in both acidic and alkaline conditions, and the amount of P release is larger in alkaline condition [13]. A previous study [20] revealed that the variation of pH can change the particles aggregation/cohesion behavior by altering their surface charge properties. The particles charge negatively, and the aggregation and sedimentation do not occur under acidic condition. The particle charge reverses from negative to positive at pH = 7, which may consider as isoelectric point. Large aggregation formed at pH = 7. In accordance with the results, the neutral condition has disadvantage of P release. The effect of pH on P release was mainly shown through the P speciation in combination with metals such as Fe, Al, and Ca [21]. The combination form of Fe-P and Al-P could exist in the sediment. At pH = 7–9, a layer of Fe(OH)_3_ protective film is formed in the surface of Fe-P, which have Fe-P stabled relatively. Moreover, the phenomenon of closed storage mechanism occurs in Al-P, and the P fraction is mainly consisted of H_2_PO_4_^−^ and H_2_PO_4_^2−^, which can be uptake by microorganisms easily. The exchange between Fe-P, Al-P, and OH^−^ easily under the higher pH.

**Figure 2:**
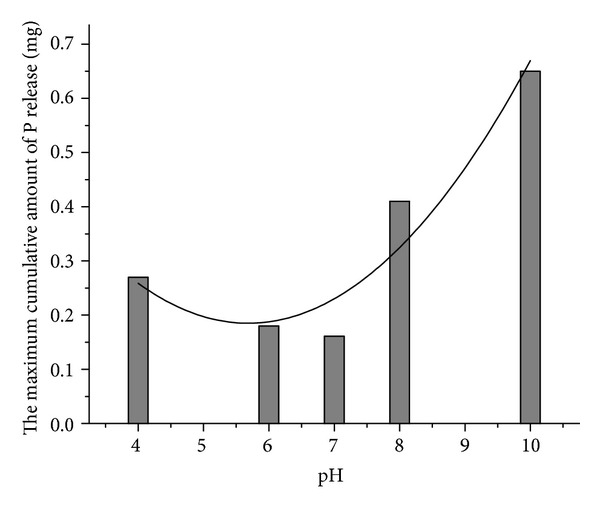
Maximum cumulative amount of P release as a function of pH.

The results indicate that the released TP reached the maximum concentration in the overlying water during the first 10 to 20 minutes of the experiment at various pH conditions. Then the concentration of TP began to decrease and finally keep equilibrium. The time to reach equilibrium was about 60 minutes at pH 4 or 6 and 30 minutes under neutral condition (i.e., pH = 7). However, in alkaline medium, the equilibrium time was much longer (i.e., 70 minutes and 80 minutes) at pH 8 and 10, respectively. It was suggested that the equilibrium time of TP concentration was the shortest under neutral condition. The P release from sewer sediments was more stable under neutral condition than other conditions. In neutral conditions, the P in the water can be consumed by some microorganism such as phosphorus-accumulating bacteria through metabolism. However, under the alkaline condition, some metal ions exist at the form of hydroxide gel or inorganic salt in the water. A certain amount of P can be adsorbed by the surface of those forms. In addition, the concentration of TP in the overlying water decreased with slow flocculation and sedimentation. The maximum cumulative amount of P release under different pH is illustrated in Figure 2. With the increase of pH, the maximum cumulative amount of P release presents “U” curve. In order to describe the relationship between the maximum cumulative amount of P release and pH, a parabolic equation was developed using the Origin 8.0 software. The equation is

(3) $$\begin{array}{c} y=0.02602x^{2}-0.29583x+1.02577,\\ R^{2}=0.84526. \end{array}$$

### 3.3. Effects of Temperature on P Release from the Sediment and Water Interface

**Figure 3:**
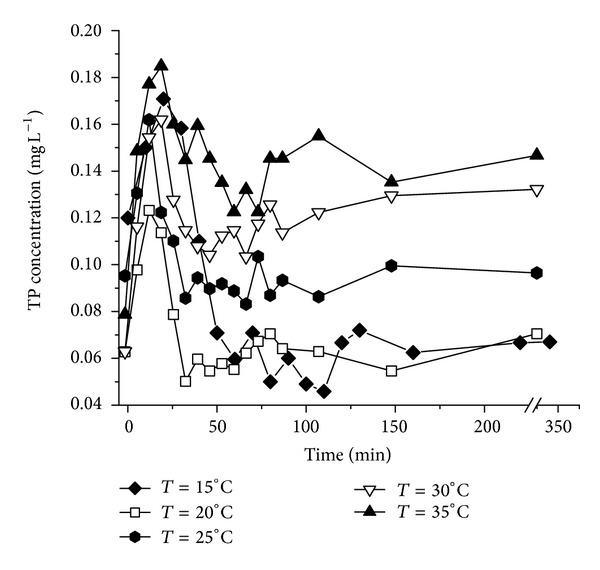
Concentration changes of TP in the temperature effect experiments.

 The effect of temperature on P release from the sediments is shown in Figure 3. Those observations suggest that P release increased with the increase of temperature. In the beginning of the experiments, the concentration of TP in overlying water increased dramatically and reached to maximum concentration in 20–30 minutes. 

 However, the concentration of TP in the overlying water decreased gradually and then tended to reach equilibrium. The equilibrium time was about 50 minutes at 15°C and 20°C, and it was about 60 minutes and 80 minutes at 25°C and 30°C, respectively. With the temperature increasing, the activity of microorganisms was increased significantly [22]. Meanwhile, the DO concentration in the overlying water decreased due to the microorganisms consumption, which would decrease the redox potential (Eh). The transformation from Fe^3+^ to Fe^2+^ was enhanced, which resulted in the release of Fe-P from the sediments [23]. In addition, the transformation from OP to IP in the sediments would be enhanced by microbial activities which would also promote the P release. The effect of P release is much significant from calcareous sediments where the mineralization of organic matter can be enhanced with the increase of temperature. A large amounts of CO_2_ was produced which results in the dissolving of calcareous sediments. The P release from sediments speeds up accordingly. Moreover, organic acids as the function of complexation can be produced in the process of organic matter decomposition, such as citric acid and tartaric acid. The release rate of P from sediments can also be enhanced by the organic acids [24].

**Figure 4:**
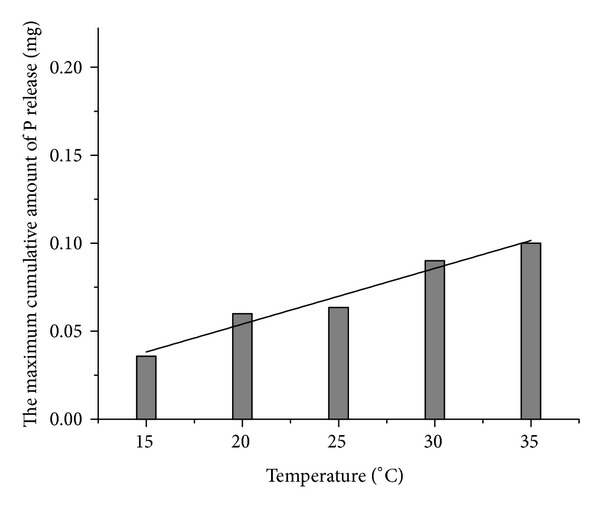
Maximum cumulative amount of P release as a function of temperature.

The maximum cumulative amount of P release under different temperature is shown in Figure 4. The maximum cumulative amount of P release increased linearly with the increase of temperature. The correlation equation is

(4) $$\begin{array}{c} y=0.00317x-0.00928,\\ R^{2}=0.94731. \end{array}$$

### 3.4. Effects of DO on P Release at the Sediment and Water Interface

**Figure 5:**
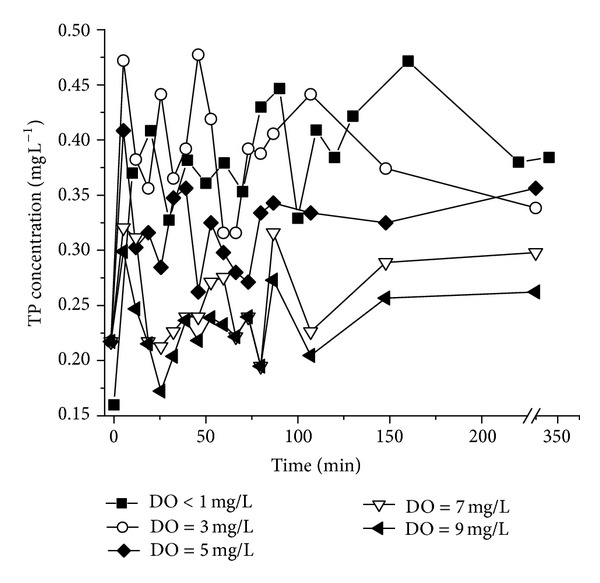
Concentration changes of TP in the DO effect experiments.

 Changes of TP concentrations of the overlying water in the release experiment are shown in Figure 5. The concentration of TP increased as DO concentration decreased. TP concentration reaches the top under anaerobic condition (DO < 1 mg L^−1^), and a much longer time (about 110 min) was needed to reach equilibrium. The similar trend is between DO < 1 mg L^−1^ and DO = 3 mg L^−1^. The TP concentration did not change too much when DO were 5 mg L^−1^, 7 mg L^−1^, and 9 mg L^−1^, where the equilibrium time was 80 min, 60 min, and 60 min, respectively.

This observation can be explained by a certain amount of particles suspended due to the injection of overlying water at experimental preparation phase. The concentration of P was then reduced by the settling and readsorption by the sediment particles. TP concentration gradually increased in anaerobic conditions with the increase of time and then tended to reach equilibrium. The equilibrium time was much longer than that in aerobic conditions. In addition, the maximum release rate of P increased as DO decreased.

**Figure 6:**
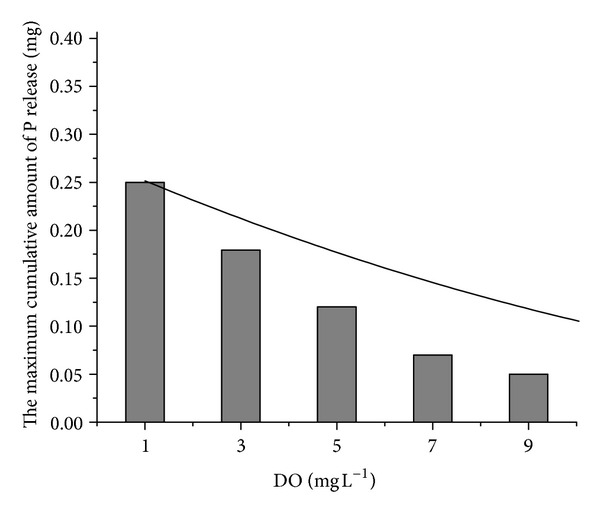
Maximum cumulative amount of P release as a function of DO.

Besides time, the Eh of sediments would also be affected by DO. The previous study [25] showed that the main release form is Fe-P. The process of chemical reactions is that Fe^3+^converts into Fe^2+^ under anaerobic conditions. The colloidal Fe(OH)_3_ protective layer on the surface of Fe-P was converted into soluble Fe(OH)_2_; therefore, PO_4_^3−^ was released from sediment into the overlying water. However, a small amount of P can be also released in aerobic conditions mainly caused by the aerobic decomposition of organic matter. The organic colloids formed were covered on the surface of inorganic solid such as clay minerals, alumina, iron oxide and calcium carbonate et al. As a result, P fixation was reduced with the reduction of the combination of PO_4_^3−^ and solids. In addition, soluble P was gradually adsorbed by Fe(OH)_3_. The relationship between the maximum cumulative amount of P release and DO is illustrated in Figure 6. The correlation equation is

(5) $$\begin{array}{c} y=0.00197x^{2}-0.0452x+0.29481,\\ R^{2}=0.99683. \end{array}$$

### 3.5. Effects of Flow Rate on P Release at the Sediment and Water Interface

**Figure 7:**
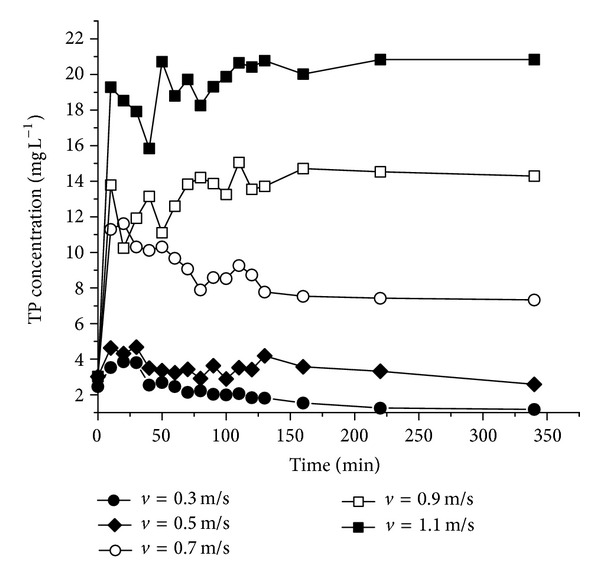
Concentration changes of TP in the flow rate effect experiments.

 The changes of TP concentration in different flow conditions with time are shown in Figure 7. It is clear that the TP concentrations in dynamic conditions are much higher than these in static conditions. The TP concentration is basically stable when the flow rate is 0.3 m s^−1^ or 0.5 m s^−1^. The TP release increased dramatically with the increase of the flow rate. The concentration of TP increased rapidly at initial stage of the experiment and then decreased gradually until it reached equilibrium. The equilibrium time increased as flow rate increased. However, the P release would not increase when the maximum quantity of P release is reached. This suggests that the influence of hydrodynamic conditions is only a short-term effect on P release.

**Figure 8:**
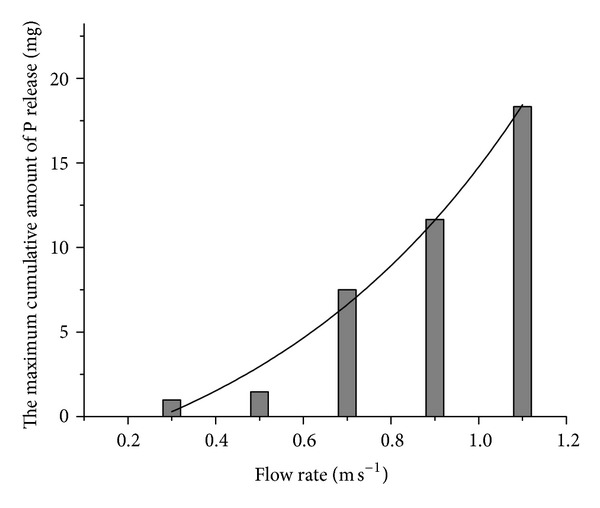
Maximum cumulative amount of P release as a function of flow rate.

In Figure 8, the maximum cumulative amount of P release increased exponentially with the increase of the flow rate. The correlation equation is

(6) $$\begin{array}{c} y=4.58073e^{1.55965x},\\ R^{2}=0.96682. \end{array}$$

According to the calculation results above, the maximum P loading is 8.29 mg m^−2^ min^−1^, 0.82 mg m^−2^ min^−1^, 2.11 mg m^−2^ min^−1^, and 178.05 mg m^−2^ min^−1^ under different factors (pH, temperature, DO and flow rate), respectively. And, it is clear that flow rate is the primary factor for P release.

## 4. Conclusions

The release rules and their affecting factors (i.e., pH, temperature, DO) of P at the sediment and water interface in storm sewer is similar to that in natural water bodies. It was almost not released in the neutral pH condition. However, the release rate of P increased with the increase of pH from 8 to 10 and is much faster at high temperature than lower. The P release was much higher in anoxic condition than that in aerobic condition.

The fitting formulas were developed to describe the relationship between the maximum cumulative amount of P release and the environmental factors in storm sewer. The P release loading in dynamic conditions are much higher than that in static conditions, flow rate is the primary affecting factor. The cumulative amount of P release increased with the process of pipeline runoff in the rainfall events with high intensities and shorter durations. Feasible measures such as best management practices and low impact development can be conducted to control P release on urban sediments by slowing down the flow rate.

## Figures and Tables

**Table 1:** Size fraction distribution.

Size fraction (mm)	<0.385	0.385–0.076	0.076–0.15	0.15–0.3	0.3–0.701	0.701–1.25	1.25–2	>2
Mass fraction (%)	0.80	2.01	3.71	10.34	29.91	17.47	12.65	23.11

**Table 2:** Various forms of P and their content distributions.

P forms	O-P	IP	OC-P	Ca-P	Fe-P Al-P	TP
Content (mg kg^−1^)	592.4	2583.5	292.8	997.9	1198.4	3071.0

Organophosphorus (O-P); inorganic phosphorus (IP); occluded phosphorus (OC-P); Calcium Phosphorus (Ca-P); iron and aluminum phosphorus (Fe-P Al-P).
